# Potential of Induced Metabolic Bioluminescence Imaging to Uncover Metabolic Effects of Antiangiogenic Therapy in Tumors

**DOI:** 10.3389/fonc.2016.00015

**Published:** 2016-02-01

**Authors:** Stefano Indraccolo, Wolfgang Mueller-Klieser

**Affiliations:** ^1^Immunology and Molecular Oncology Unit, Istituto Oncologico Veneto – IRCCS, Padova, Italy; ^2^University Medical Center of the Johannes Gutenberg University, Institute of Pathophysiology, Mainz, Germany

**Keywords:** angiogenesis, metabolism, imaging, glycolysis, cancer, mouse models

## Abstract

Tumor heterogeneity at the genetic level has been illustrated by a multitude of studies on the genomics of cancer, but whether tumors can be heterogeneous at the metabolic level is an issue that has been less systematically investigated so far. A burning-related question is whether the metabolic features of tumors can change either following natural tumor progression (i.e., in primary tumors versus metastasis) or therapeutic interventions. In this regard, recent findings by independent teams indicate that antiangiogenic drugs cause metabolic perturbations in tumors as well as metabolic adaptations associated with increased malignancy. Induced metabolic bioluminescence imaging (imBI) is an imaging technique that enables detection of key metabolites associated with glycolysis, including lactate, glucose, pyruvate, and ATP in tumor sections. Signals captured by imBI can be used to visualize the topographic distribution of these metabolites and quantify their absolute amount. imBI can be very useful for metabolic classification of tumors as well as to track metabolic changes in the glycolytic pathway associated with certain therapies. Imaging of the metabolic changes induced by antiangiogenic drugs in tumors by imBI or other emerging technologies is a valuable tool to uncover molecular sensors engaged by metabolic stress and offers an opportunity to understand how metabolism-based approaches could improve cancer therapy.

## Introduction

Malignant tumors are endowed with distinct metabolic features that distinguish them from the normal cells of the tissue where they arise. Major metabolic alterations in tumors include enhanced glucose uptake and lactate production, increased glutamine utilization and *de novo* fatty acid synthesis, as well as aberrant choline and serine metabolism (1, 2). Tumor heterogeneity – best known at the genetic level – has recently been illustrated also at the metabolic level by a number of different approaches (3, 4). Still largely unknown is whether metabolic features of tumors are stable or can change either following natural tumor progression (i.e., in primary tumors versus metastasis) or therapeutic interventions.

Antiangiogenic therapy is a consolidated approach to treat cancer. By targeting the tumor-associated microvasculature, antiangiogenic drugs – especially blocking signaling downstream of vascular endothelial growth factor (VEGF)/VEGF receptors – cause vessel pruning, which is followed by hypoxia and reduced supply of other nutrients (5–8). Since antiangiogenic drugs do not have, in general, direct effects of tumor cells; the therapeutic activity of these drugs is almost entirely dependent on modulation of the supportive functions of the tumor microenvironment. Albeit still hypothetical, it is quite possible that the ability of tumor cells to adapt to the starving effects of antiangiogenic therapy – i.e., its metabolic effects – could be important to determine the therapeutic activity of these drugs.

Imaging techniques are extremely valuable both to perform metabolic profiling of tumors and to uncover metabolic fluctuations caused by drugs. With regard to antiangiogenic therapy, some of these techniques have been used to measure uptake of selected metabolites in tumors, such as positron emission tomography (PET) (9), whereas others, including hyperpolarized ^13^C spectroscopy (10) and proton nuclear magnetic resonance (NMR) (11), enabled quantitative assessment of certain metabolites in experimental tumors.

We were the first to exploit a technique termed induced metabolic bioluminescence imaging (imBI) to investigate metabolic perturbations associated with anti-VEGF therapy (9, 12). Notably, although somewhat limited by the small number of metabolites that can be analyzed, imBI provides an accurate representation of the steady-state levels of the metabolites, matching them to the histology of the tumor section. The rest of this chapter is devoted to the methodologic description of this valuable imaging technique and its applications in the field of tumor angiogenesis.

## The Technique for Induced Metabolic Bioluminescence Imaging

### Specific Detection of Tissue Metabolites

The imBI technique was developed in the laboratory at the University of Mainz for the detection of metabolites in cryosections from snap-frozen tissue *in situ* (13–15). Self-made kits containing definite exogenous enzymes and cofactors are used to achieve high specificity and sensitivity. Endogenous enzyme activities in the tissue sections of interest are heat-inactivated immediately before imBI measurement to avoid interference with exogenous enzymes within the kit. To prevent the reactions from substrate saturation, all components of the kit are provided in excess. The enzymatic activity induced by the metabolite of interest in the target tissue is biochemically coupled to luciferases leading to light emission, i.e., to bioluminescence. This coupling, which involves the NAD(P)H^+^H^+^ redox system, induces low level light emission with an intensity that is proportional to the respective metabolite. The biochemistry for the detection of ATP, glucose, pyruvate, and lactate *via* imBI is illustrated diagrammatically in Figure S1 in Supplementary Material. Since a specific enzyme mixture has to be prepared for each metabolite of interest, the imBI technique allows for the detection of only one metabolite per section. On the other hand, section thickness is adjusted in most cases to values in the range of 10–20 μm, and thus different metabolites can be determined in serial sections at a quasi-identical location within a tissue.

**Figure 1:**
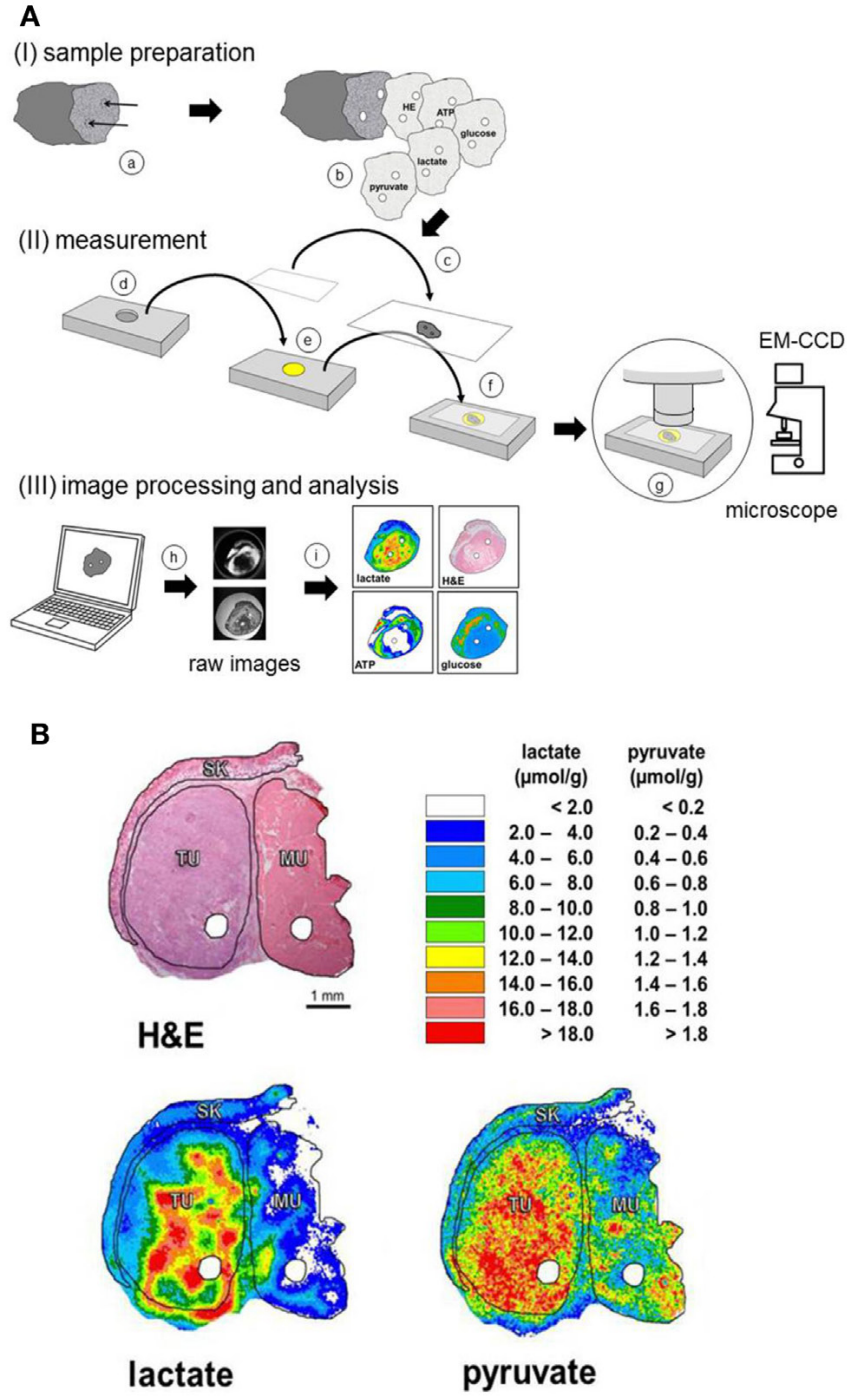
**Schematic of the procedure of induced metabolic bioluminescence imaging (imBI) and representative pictures from a human tumor sample**. **(A)** Schematic of the procedure of induced metabolic bioluminescence imaging (imBI). (I) Sample preparation: the frozen tissue sample is precisely punctured by a small fork (a). Subsequently, serial cryosections are prepared (b). Each section is adhered on a cover glass (c) and heat inactivated (not shown). (II) A casting mold (d) is filled with an appropriate enzyme solution (e). Immediately before measurement, a frozen section on a cover glass is immersed into the enzyme solution (f). This “sandwich” is inserted into a thermostated chamber mounted on the stage of a microscope (g). The light emission is registered by an EM-CCD-camera connected to a computer. (III) Using an image analysis software (h), the raw images are processed, analyzed, calibrated, and, finally, expressed as color-coded images (i). Pinpricks (a) within the biopsy provide landmarks for a correct overlay of corresponding images. **(B)** Induced metabolic bioluminescence imaging (imBI) of lactate and pyruvate in a human head and neck squamous cell carcinoma (HNSCC) xenografted in a nude mouse. Sequential cryosections (each showing two punched holes for overlay) were used for hematoxylin and eosin (H&E) staining and imaging of lactate and pyruvate concentrations. For structure-associated evaluation, defined histological areas were delineated: tumor tissue (TU), skeletal muscle (MU), and skin (SK) [modified according to Walenta et al. (15)].

For processing of the tissue specimens, the frozen biopsy is embedded in Tissue-Tek^®^ (O.C.T.™ Compound; Sakura Finetek Europe B.V., Alphen aan den Rijn, Netherlands) on a sample holder of a cryomicrotome (SLEE cryostat, Type MEV; SLEE, Mainz, Germany). Serial sectioning is then performed precisely at a temperature between −20 and −12°C depending on the tissue composition of the specimen. As indicated in the upper panel of Figure 1A, two parallel channels are driven into each biopsy by a specially designed “micro-fork.” Sectioning perpendicular to these channels leads to two holes in each section. These holes make it possible to precisely overlay sequential sections and, thus, to colocalize different parameter values in different adjacent sections.

### Localization of Tissue Metabolites

The spatial resolution of imBI is generated by a sandwich technique providing a homogeneous contact between enzyme cocktail and tissue section (Figure 1A). An excess volume of enzyme solution is pipetted into a casting mold within a metal slide. A cover glass with a tissue section adhered is laid upside-down onto the enzyme solution within the casting mold. This provides complete contact between tissue and enzyme solution, which has been kept in a liquid state at the measuring temperature. The contact between substrate and enzyme mixture then initiates the bioluminescence reaction. The sandwich is put on the thermostated stage of a microscope (Axiophot, Zeiss, Oberkochen, Germany) within a light-impervious chamber. An ultrasensitive back-illuminated EM-CCD camera (iXonEM^+^ DU-888; Andor Technology PLC, Belfast, UK) connected to the microscope enables the registration of the low light bioluminescence intensity with the optics focused on the tissue section plane. The image signal is transferred to a computer for image analysis. By integrating the light emission intensities over a selected time interval, a two-dimensional density profile is obtained representing the two-dimensional metabolite distribution across the tissue section.

### Quantification of Tissue Metabolites

Using appropriate standards, which are handled in exactly the same way as the tissue of interest, bioluminescence intensities can be transformed into absolute tissue concentrations of the respective metabolite, e.g., in micromoles per gram of tissue, which corresponds approximately to millimolar in solution. Routine standards are obtained by mixing cryo-embedding medium (Tissue-Tek^®^) with defined amounts of substrate. The frozen medium is then cryosectioned and processed in the same way as the tissue of interest.

The calibration allows for the illustration of the two-dimensional substrate distribution in a color-coded manner, as indicated in Figure 1A (lower panel). This procedure is applicable to all metabolites specified above.

It is obvious that the light intensity is also influenced by the microscopic magnification, since pixel density per unit image area and, hence, light intensity is much lower at a high magnification and *vice versa*. Other factors with impact on light intensity are the viscosity of the enzyme solution, the measuring temperature, the light integration interval, or the quantum efficiency of the bioluminescence reaction itself. And all these factors are mutually related and not at all independent of each other. In essence, the concert of these factors determines the sensitivity of the technique being on the micromolar level.

### Combined Quantification and Localization of Metabolites

High sensitivity and high spatial resolution are contradicting demands, but the advantage of imBI is its versatility in adjusting the measuring conditions toward either resolution or sensitivity depending on the requirements of an actual experiment. Spatial resolution is restricted at a low viscosity and high temperature of the enzyme solution and at long light integration interval. Such conditions favor lateral diffusion of metabolites and enzymes resulting in a “smearing out” the gradients in the metabolic profiles.

For most measurements in tumor biopsies, experimental settings are chosen in a way that the minimum detectable substrate concentration is around 100 μmol/g with a spatial linear resolution of around 100 μm. Keeping the former sensitivity of detection, the spatial resolution can be adjusted to 20 μm in imaging metabolic gradients in multicellular spheroids adjusting an appropriate registration temperature and viscosity of the enzyme solution.

In our routine technology, as described above, the minimum detectable substrate concentration is around 100 μmol/g of viable tissue. Taking a given thickness of the cryosection used of 10 μm and a section area of 1 cm^2^, this corresponds to a minimum detectable absolute amount of substrate (i.e., the substrate content of one cryosection) of around 10 pg. By reducing the section thickness, increasing the enzyme components in the detection cocktails and optimizing the photon registration technique, it is possible to detect minimum substrate concentrations in the nanomolar per gram range.

### Structure-Associated Metabolite Detection and Colocalization

Using serial sections, a section can be stained for the histological structure, e.g., with hematoxylin and eosin (H&E) followed by sections stained for the various metabolites. As outlined above, the “two holes technique” allows for an exact, computer-assisted overlay between the sections. Such an overlay makes it possible to detect the bioluminescence signals in a structure-associated way, i.e., within selected histological areas, such as viable tumor regions, stromal areas, or necrosis. The strategy is exemplified in Figure 1B.

Colocalization studies can be extended to virtually all parameters that can be visualized in cryosections, e.g., mRNAs detected by *in situ* hybridization, proteins assessed by immunostaining or functional parameters, such as blood perfusion imaged by fluorescent stains or hypoxic cells identified by pimonidazole (16).

## Applications of imBI in the Field of Tumor Angiogenesis

**Figure 2:**
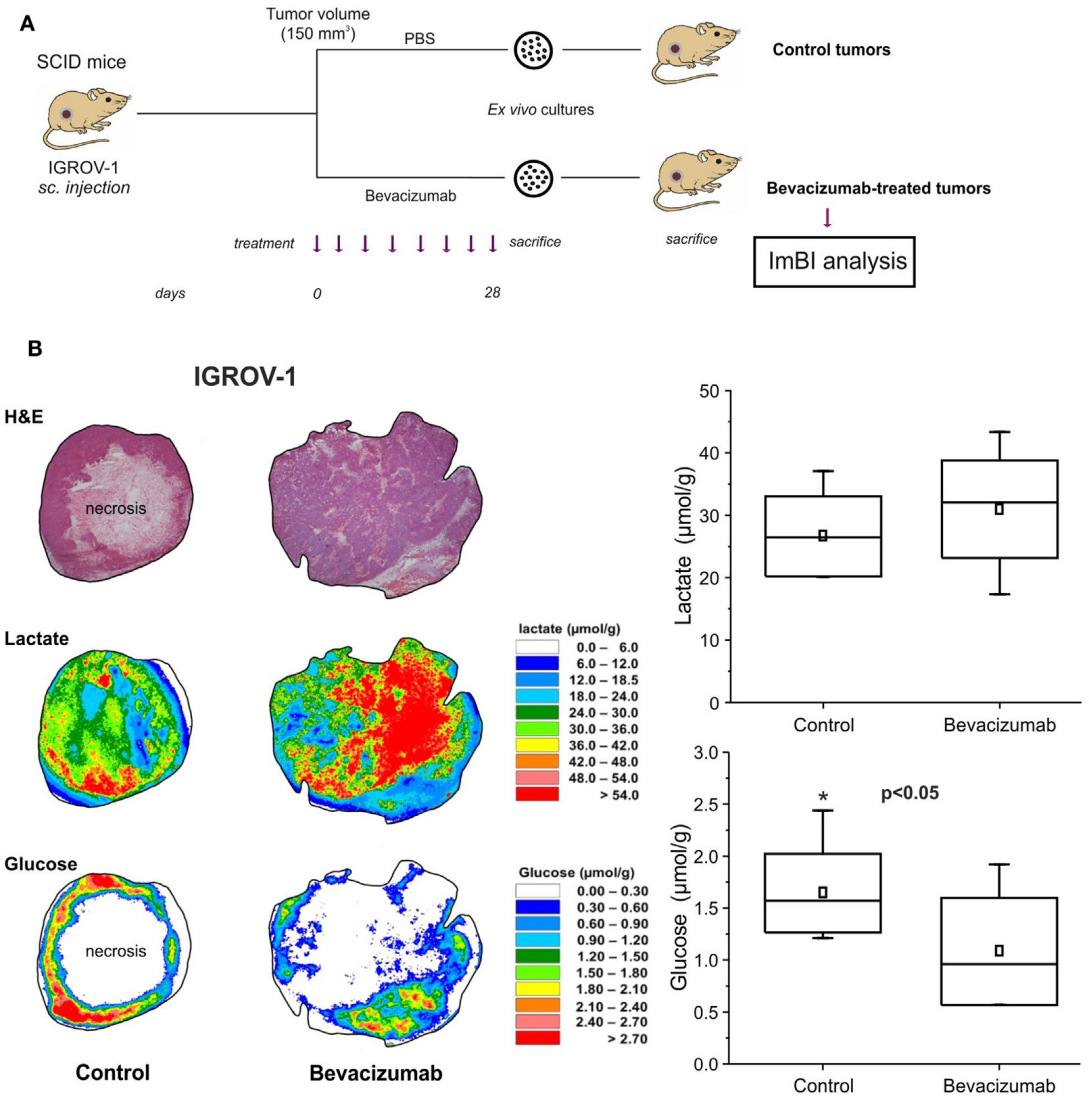
**Tumor xenografts from anti-VEGF-treated mice disclose an highly glycolytic phenotype by imBI analysis**. **(A)** Experimental layout. The tumor samples subjected to imBI analysis were obtained from secondary recipient mice injected with ovarian cancer cells (IGROV-1) of primary cultures from control or bevacizumab-treated tumors. **(B)** Quantification of lactate and glucose levels in tumor xenografts by imBI. *Left panel:* H&E staining and color-coded distributions of lactate and glucose in sequential cryosections from representative tumors. Since some tumors contain large central necrosis, glucose was at background levels within these large central areas. However, high glucose concentrations are clearly seen in the tumor periphery. This nicely illustrates the importance of structure-associated data acquisition with imBI. The concentration values were color coded, with each color corresponding to a defined concentration range in micromolar per gram. *Right panel:* metabolite concentrations of lactate and glucose in tumors. Lactate and glucose values were calculated from *n* = 9 and *n* = 7 sections from anti-VEGF-treated or control tumors, respectively [reproduced from Curtarello et al. (9)].

Induced metabolic bioluminescence imaging proved to be an extremely useful technique to investigate modulations in the concentration of metabolites in tumors treated with antiangiogenic drugs (Figure 2). In tumor xenografts, we observed a dramatic reduction in glucose and ATP levels in the tumor microenvironment following anti-VEGF therapy (12). Such changes led us to speculate that glucose-addicted tumors could be particularly vulnerable to the effects of VEGF blockade. In fact, the amount of necrotic area in the highly glycolytic tumors was much larger compared with values found in poorly glycolytic tumors, thus connecting a metabolic trait of tumor cells to an histological pattern of response to VEGF neutralization.

It is currently unknown whether this intriguing observation might hold true also in the case of other angiogenesis inhibitors acting downstream of the VEGF receptor, such as tyrosine kinase inhibitors, nor whether the findings can be generalized to tumors grown in different organs/tissues, as tissue-specific features of the microvasculature could also impact on the outcome of VEGF blockade. Despite these limitations, it should be acknowledged that imBI was key to enable us to formulate the initial hypothesis, To further validate it in translational studies, it will probably be necessary to replace assessment of the tumor gylcolytic phenotype by imBI – which requires frozen tissues – with an appropriate *in situ* marker of glycolysis, such as monocarboxylate transporter 4 (MCT4), which could be assessed by routine immunohistochemistry staining in hundreds of formalin-fixed paraffin-embedded (FFPE) tumor samples.

In addition, imBI could be used to perform metabolic classification of tumors and compare *in vitro* and *in vivo* assessment of metabolic features, as we recently showed (17). Given the adequate spatial resolution of this technique (~100 μm), and the possibility of matching metabolic parameters with histology, it can be anticipated that imBI could be utilized to describe both inter-tumor and intra-tumor metabolic heterogeneity, as first step of an in-depth characterization of tumor regions with different abundance of lactate or other measurable metabolites.

In summary, imBI has a clear role in preclinical studies of tumor angiogenesis in that it can (I) be utilized to investigate metabolic modulations associated with antiangiogenic therapy and (II) can enable metabolic classification of tumors and detect metabolic heterogeneity.

## Concluding Remarks

A more comprehensive understanding of metabolic perturbations caused by antiangiogenic drugs in tumors might represent a first step toward improvement of therapeutic efficacy, which is currently blunted by adaptive resistance (18, 19). Along this line, recent studies reported perturbations in lipid metabolism under hypoxic conditions. Bensaad et al. described increased fatty acids uptake and accumulation of lipid droplets in the cytoplasm of tumor cells incubated under hypoxic conditions or in tumors treated with bevacizumab (20); this phenomenon was associated with hypoxia inducible factor (HIF)-1α-driven expression of fatty acids binding proteins 3 and 7 in tumor cells. Moreover, Huang et al. observed that hypoxic stress regulates lipid metabolism in hepatocellular carcinoma cells. Specifically, these authors reported that HIF-1-mediated suppression of medium and long-chain acyl-CoA dehydrogenases and fatty acid oxidation leads to fatty acids accumulation and is critical for cancer progression (21). As antiangiogenic therapy generally increases tumor hypoxia, in future studies, it will be important to further investigate whether anti-VEGF therapy causes perturbations in lipids.

In the long-term, metabolic changes caused by anti-VEGF therapy could indeed represent an opportunity for new metabolism-targeting drugs. In this regard, our previous studies (9, 12) showed that tumor xenografts formed by different tumor cell lines have marked metabolic differences, which would likely convey limited therapeutic response to glycolysis inhibitors (Figure 2). In contrast, after antiangiogenic therapy tumors become homogeneously highly glycolytic and glucose addicted, which could render them more responsive to glycolysis-targeting drugs. In a related study, Sounni et al. demonstrated that breast tumors treated with antiangiogenic tyrosine kinase inhibitors (sunitinib) in the post-treatment time undergo a metabolic shift involving increased lipid synthesis and have improved response to the lipogenesis inhibitor orlistat (22). Altogether, these observations suggest that sequential combination of antiangiogenic drugs and certain metabolism-targeting drugs might represent a new therapeutic concept for cancer.

With regard to imBI, its successful application in the field of glucose metabolism should encourage set-up of new protocols for the detection of additional metabolites, such as amino acids and fatty acids. The imBI technique is indeed appropriate for the detection of any metabolite that can be enzymatically linked to the NAD(P)H^+^H^+^ redox system. This offers the opportunity to analyze a broad spectrum of metabolic pathways beyond glycolysis. For example, lipid metabolism may be investigated by quantifying l-3-hydroxyacyl-CoA with imBI using hydroxyacyl-CoA-dehydrogenase as an enzymatic link. Similarly, ketogenesis may be studied by the detection of acetoacetate using beta-hydroxybutyrate dehydrogenase.

In conclusion, in the near future, it will be feasible to use imBI to achieve an integrated view on serial tumor sections of the metabolic pathways active in individual tumors and their variations following targeted therapies.

## Author Contributions

SI and WM-K wrote and edited the text.

## Conflict of Interest Statement

The authors declare that the research was conducted in the absence of any commercial or financial relationships that could be construed as a potential conflict of interest.

## Abbreviations

H&E, hematoxylin and eosin; HIF, hypoxia inducible factor; imBI, induced metabolic bioluminescence imaging; NMR, nuclear magnetic resonance; PET, positron emission tomography; VEGF, vascular endothelial growth factor.
