# Supplementary material for: Potential of Induced Metabolic Bioluminescence Imaging to Uncover Metabolic Effects of Antiangiogenic Therapy in Tumors
Source: Front Oncol. 2016 Feb 1;6:15. doi: 10.3389/fonc.2016.00015 (PMC4733917; doi:10.3389/fonc.2016.00015)
Supplement: Supplementary file 1 [file Image_1.PDF]

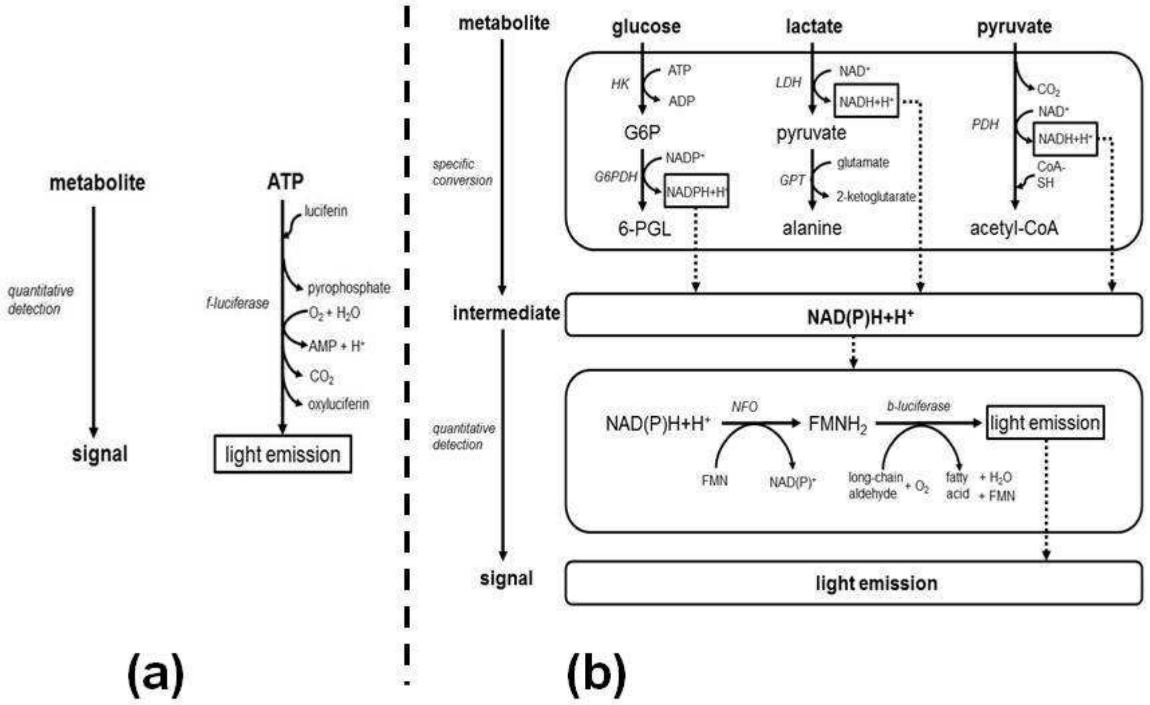

Suppl. Figure 1: Schematic of the biochemistry of induced metabolic bioluminescence imaging (imBI):

(a) ATP immediately takes part in the bioluminescence reaction of fireflies (*Photinus pyralis*).

(b) Glucose, lactate and pyruvate are enzymatically converted to a common intermediate metabolite, NADH + H^+ or NADPH + H^+. Subsequently, the coenzyme enters the bioluminescence reactions of *Photobacterium fischeri* yielding light emission proportional to the initial metabolite concentration. Asterisk: excited state.

(Modified according to (Walenta, 2014))

Abbrevations:

Enzymes: hexokinase (HK), glucose-6-phosphate-dehydrogenase (G6PDH), lactate dehydrogenase (LDH), glucose-pyruvate-transaminase (GPT), pyruvate-dehydrogenase (PDH), NAD(P)H:FMN-oxidoreductase (NFO), bacterial luciferase (b-luciferase), firefly luciferase (f-luciferase). Metabolites: glucose-6-phosphate (G6P), 6-phospho-gluconolactone (6-PGL).
